# Genetic effect of interleukin-1 beta (C-511T) polymorphism on the structural covariance network and white matter integrity in Alzheimer’s disease

**DOI:** 10.1186/s12974-017-0791-z

**Published:** 2017-01-18

**Authors:** Chi-Wei Huang, Shih-Wei Hsu, Shih-Jen Tsai, Nai-Ching Chen, Mu-En Liu, Chen-Chang Lee, Shu-Hua Huang, Weng-Neng Chang, Ya-Ting Chang, Wan-Chen Tsai, Chiung-Chih Chang

**Affiliations:** 1 Department of Neurology, Cognition and Aging Center, Kaohsiung Chang Gung Memorial Hospital, Chang Gung University College of Medicine, #123, Ta-Pei Road, Niaosung, Kaohsiung County 833 Taiwan; 2 Department of Radiology, Kaohsiung Chang Gung Memorial Hospital, Chang Gung University College of Medicine, Niaosung, Kaohsiung Taiwan; 3 Psychiatric Department of Taipei Veterans General Hospital, Taipei, Taiwan; 4 Psychiatric Division, School of Medicine, National Yang-Ming University, Taipei, Taiwan; 5 Department of Nuclear Medicine, Cognition and Aging Center, Kaohsiung Chang Gung Memorial Hospital, Chang Gung University College of Medicine, Niaosung, Kaohsiung Taiwan

**Keywords:** Alzheimer’s disease, Anatomical structural covariance, Interleukin-1 beta polymorphism, Salience network, Executive control network

## Abstract

**Background:**

Inflammatory processes play a pivotal role in the degenerative process of Alzheimer’s disease. In humans, a biallelic (C/T) polymorphism in the promoter region (position-511) (rs16944) of the interleukin-1 beta gene has been significantly associated with differences in the secretory capacity of interleukin-1 beta. In this study, we investigated whether this functional polymorphism mediates the brain networks in patients with Alzheimer’s disease.

**Methods:**

We enrolled a total of 135 patients with Alzheimer’s disease (65 males, 70 females), and investigated their gray matter structural covariance networks using 3D T1 magnetic resonance imaging and their white matter macro-structural integrities using fractional anisotropy. The patients were classified into two genotype groups: C-carriers (*n* = 108) and TT-carriers (*n* = 27), and the structural covariance networks were constructed using seed-based analysis focusing on the default mode network medial temporal or dorsal medial subsystem, salience network and executive control network. Neurobehavioral scores were used as the major outcome factors for clinical correlations.

**Results:**

There were no differences between the two genotype groups in the cognitive test scores, seed, or peak cluster volumes and white matter fractional anisotropy. The covariance strength showing C-carriers > TT-carriers was the entorhinal-cingulum axis. There were two peak clusters (Brodmann 6 and 10) in the salience network and four peak clusters (superior prefrontal, precentral, fusiform, and temporal) in the executive control network that showed C-carriers < TT-carriers in covariance strength. The salience network and executive control network peak clusters in the TT group and the default mode network peak clusters in the C-carriers strongly predicted the cognitive test scores.

**Conclusions:**

Interleukin-1 beta C-511 T polymorphism modulates the structural covariance strength on the anterior brain network and entorhinal-interconnected network which were independent of the white matter tract integrity. Depending on the specific C-511 T genotype, different network clusters could predict the cognitive tests.

**Electronic supplementary material:**

The online version of this article (doi:10.1186/s12974-017-0791-z) contains supplementary material, which is available to authorized users.

## Background

The neuropathology of Alzheimer’s disease (AD) involves a profound innate immune response and production of inflammatory cytokines [1]. Whether or not inflammatory processes represent a causal component of AD or are simply a consequence of neurodegeneration is still under debate. However, it is generally accepted that inflammatory cascades [2] together with neurofibrillary tangles [3] and degenerative neuroimaging biomarkers [4] are more strongly associated with cognitive declines in patients with AD. Many inflammatory mediators have been identified in the brains of patients with AD. Variations in inflammatory-related genes have been extensively investigated [5–8], and the expression of interleukin-1 (IL-1) beta has been shown to have clinical relevance. IL-1 beta, produced in the glial cells and neurons, has been reported to have a higher density in the hippocampus in response to stress of injury [9]. In addition, in an Alzheimer’s mouse model, IL-1 beta-related inflammatory responses surprisingly reduced amyloid beta deposition [10] but paradoxically enhanced tau pathology [11].

A large number of functional polymorphisms in the promoter regions of pro- and anti-inflammatory genes have been associated with different levels of mediators that have an overall effect on the strength of inflammatory responses [12]. The human interleukin-1 beta gene (IL1B) is located on chromosome 2q14. A biallelic (C/T) polymorphism in the promoter region (position-511) (rs16944) of the IL-1 beta gene has been significantly associated with IL-1 beta secretory capacity after lipopolysaccharide-stimulation that T homozygous individuals secreted significantly more IL-1 beta than CC and CT individuals [13]. In Taiwan, the −511 TT genotype has also been associated with an increased risk of AD [14]. The substantial increase in the expression of the IL-1 beta gene during long-term potentiation of synaptic transmission suggests its role in synaptic function [15]; however, blocking of IL-1 beta in the hippocampus has been reported to produce significant memory impairment compared with vehicle-treated rats [16].

Both deep and periventricular white matter hyperintensities (WMHs) have been reported to be significantly associated with AD degeneration process in both cross-sectional and longitudinal studies [17–19]. The presence of WMHs in AD may reflect multiple physiological and pathological changes such as breakdown of the blood-brain barrier [20], impaired cerebral auto-regulation [21], vasculopathies, and inflammation [22, 23]. In healthy adults, homozygotes for the IL-1 beta -511 T allele have been associated with increased inflammatory responses and larger WMHs than the other two genotypic combinations [24]. The -511 T homozygotes are also associated with a higher risk of ischemic stroke, suggesting a role in large [25] or small vessel pathology [26]. These observations make the IL-1 beta C-511 T genetic variant a plausible candidate to evaluate the genetic effect on WMH proliferation. Data on the C-511 T with regards to WMH load and gray matter (GM) degeneration in AD are still lacking.

The application of structural covariance networks (SCNs) has been supported by recent research in that highly related regions may show covariance in morphometric characteristics. SCN patterns have been shown to be associated with structural or functional connectivity while the structural covariance strength often reflects how close two interconnected hubs interact [27]. Meanwhile, biological factors such as genetic variations, developmental, degenerative processes or the WMH loads [24] have been shown to modulate the patterns of SCNs [27]. Three SCNs have been reported to be relevant to AD, the so called default mode network (DMN) [28–30], salience network (SN) [31], and executive control network (ECN) [32, 33]. A recent report suggested that the DMN may be comprised of multiple, spatially dissociated but interactive components, of which two subsystems are particularly relevant: the “medial temporal lobe subsystem”, and the “dorsal medial prefrontal cortex subsystem” (or the midline core subsystem) [34].

An association between the IL-1 beta -511 T allele and AD has been reported [14], however the mechanism by which IL-1 beta functional polymorphisms affect the brain networks in patients with AD has yet to be explored. Recent neuroscience studies have supported that cognitive function is highly reflective of the architecture of the neuronal network scaffold [35]. As the potential mechanisms of genetic-based neurobiology are still under investigation [36–39], the exploration of IL-1 beta functional polymorphism may address how genetic variations may affect the organization of the GM and white matter (WM) integrities. The neuroimaging biomarkers of SCN and WMH spatial distribution can be used to test the influence of the genotype groups with regards to inflammatory processes on disease-related degeneration. In this study, we hypothesized that the IL-1 beta C-511 T functional polymorphism may modulate large-scale structural covariance patterns and WMHs in patients with AD, and that these alterations may consequently determine neurobehavioral performance.

## Methods

The patients were treated at the Cognition and Aging Center, Department of General Neurology, Kaohsiung Chang Gung Memorial Hospital. A total of 135 AD subjects (65 males, 70 females) were included after the consensus of a panel composed of neurologists, neuropsychologists, neuroradiologists, and experts in nuclear medicine [23]. AD was diagnosed according to the International Working Group criteria [40], with a clinical diagnosis of typical AD encompassing both prodromal and dementia phases. Amnestic syndrome of the hippocampal type was characterized by a low free recall that was not normalized by cueing [41]. All of the patients had a clinical dementia rating scores ranges from 0.5 to 2, and all of the patients were in a stable condition under treatment with acetylcholine esterase inhibitors from the time of diagnosis. The exclusion criteria were a past history of clinical stroke, a modified Hachinski ischemic score >4 [42] and depression.

### Study working scheme

Based on the study rationale, the patients were classified into two genotype groups based on the functional polymorphism: C carrier (CC + CT, *n* = 108), and TT homozygotes (*n* = 27). The working scheme was as follows. First, four SCN were established by seed-based correlation analysis. Differences in each seed regional volume and SCN peak cluster volume were compared between the two genotype groups. Meanwhile, neuroimaging biomarkers used to assess the micro-structural of the WM integrities were derived from the diffusion tensor imaging, compared between groups and correlated with cognitive test scores. Finally, to evaluate the genetic effects on SCN clusters, the covariance strength showing significant genotype interactions (i.e., T homozygotes > C-carriers or T homozygotes < C-carriers) were modeled. The volumes of the peak clusters showing genotype effect was correlated with cognitive test scores to evaluate the clinical significance.

### Clinical and neurobehavioral assessments

After enrolment, demographic data of each patient were recorded. A trained neuro-psychologist administered the neurobehavior tests. The Mini-Mental State Examination (MMSE) [43] and the Cognitive Abilities Screening Instrument (CASI) [44] total scores were used as a global assessment of cognitive function. Attention, verbal fluency, abstract thinking, and mental manipulation sub-domain scores of the CASI were used to assess executive function test (EFT) [22], while the non-executive domains included orientation, short- and long-term memory, language ability, and drawing.

### Genotyping

Genomic DNA was extracted from blood samples using a commercial kit (Qiagen, Gentra Puregene Blood Kit), followed by genotyping for C-511 T polymorphisms of the IL-1 beta gene using the polymerase chain reaction (PCR)-restriction fragment length polymorphism method [45]. Genotyping was conducted with the operator blinded to the clinical data. The apolipoprotein E genotype was also determined using a PCR-restriction fragment length polymorphism assay and restriction enzyme HhaI [46]. Apolipoprotein E4 carriers were defined as those with one or two E4 alleles [23].

### Cerebrovascular risk confounders

Factors such as oxidative stress, deregulated metabolic factors and an elevated blood sugar level are related to greater WMHs loads in AD [23]. Therefore, the following risk confounders were included for comparisons: age, high sensitive C-reactive protein, homocysteine, total cholesterol, triglyceride, high-density lipoprotein, low-density lipoprotein, creatinine, vitamin B12, folate, and hemoglobin-A1C [47].

### Image acquisition

MR images were acquired using a 3.0 T MRI scanner (Excite, GE Medical Systems, Milwaukee, WI, USA). Structural images were acquired for SCN constructions using the following protocols: a T1-weighted, inversion-recovery-prepared, three-dimensional, gradient-recalled acquisition in a steady-state sequence with a repetition time/echo time/inversion time of 8600 ms/minimal/450 ms, a 256 × 256 mm field of view, and a 1-mm slice sagittal thickness with a resolution of 0.5 × 0.5 × 1 mm3.

The diffusion-tensor imaging was acquired using the following parameters: repetition time/echo time/flip angle = 9600 ms/62.7 ms/90°, a 192 × 192 mm field of view, a 128 × 128 matrix and a 4-mm axial slice thickness. For whole brain coverage, 40 contiguous axial slices were obtained. The diffusion-weighting gradients were applied in 61 non-collinear directions, optimised by the static electron-repulsion model. The *b* value used was 1000 s/mm2. One reference image was acquired using the same imaging parameters but without diffusion weighting.

### Data analysis for neuroimaging biomarkers

#### SCN analysis

Image preprocessing and statistical analysis were performed using SPM8 (SPM8, Wellcome Trust Centre of Cognitive Neurology, University College London, UK, http://www.fil.ion.ucl.ac.uk/spm/). The T1 images were reoriented, realigned, and normalized using the standard Montreal Neurological Institute (MNI) space. The images were then segmented into GM and WM. Related tissue segments were used to create a custom template using the diffeomorphic anatomical registration using exponentiated lie algebra (DARTEL) approach that represented one of the highest ranking registration methods in patients with AD [48]. The modulated and warped images were then smoothed using a Gaussian kernel of 8 mm full width at half maximum.

**Fig. 1:**
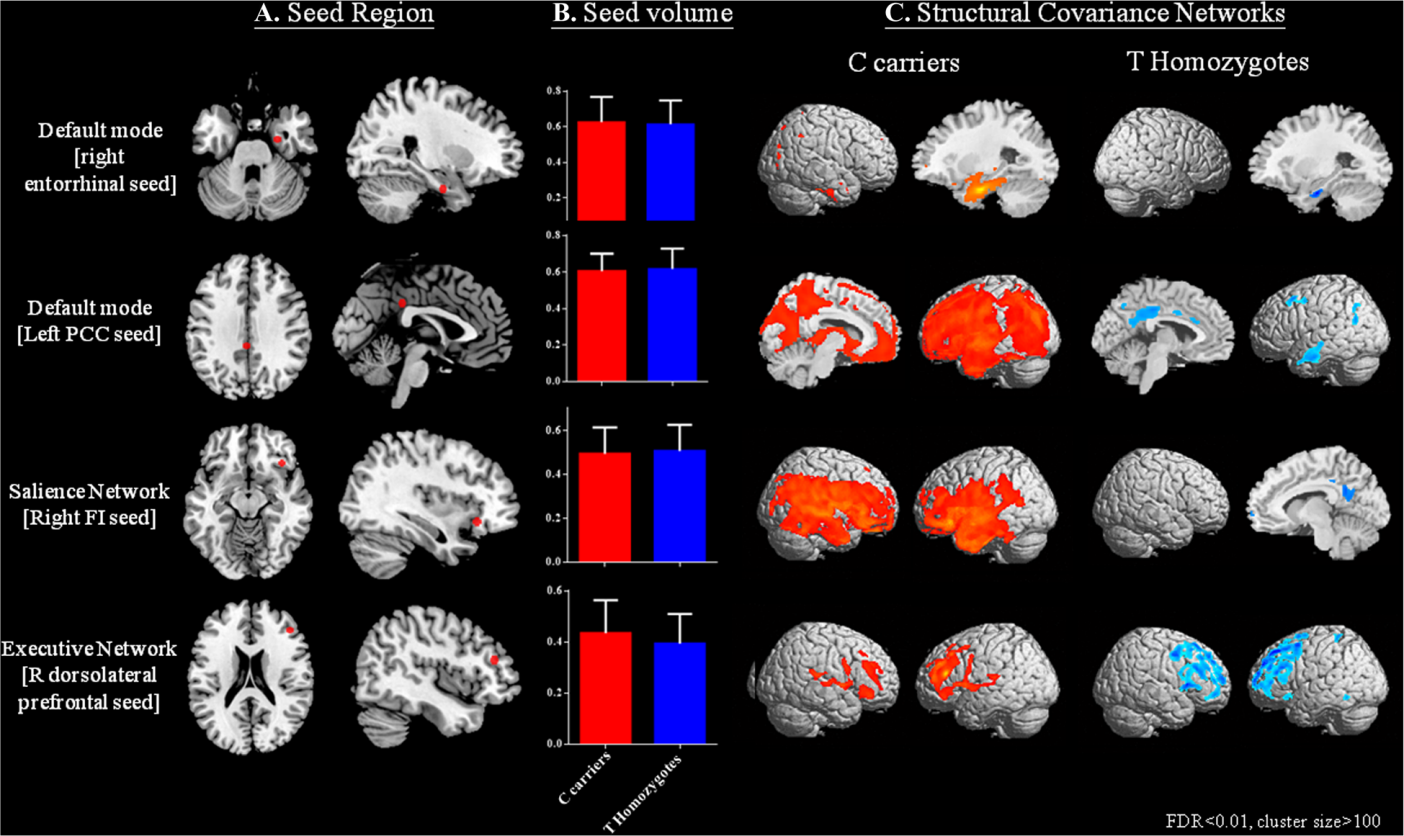
Statistical maps depicting brain areas in which the gray matter intensity covaried with (**a**) four target seeds, (**b**) comparisons of seed volumes, and (**c**) separate structural covariance network in patients with Alzheimer’s disease with interleukin-1 beta genotypes (C carrier *n* = 108, T homozygotes *n* = 27). There was no significant difference in seed volume between the genotype groups (*p* > 0.05). Z-statistic maps (*p* < 0.05, corrected with a false discovery rate with extended cluster voxels >100) are displayed on a standard brain render

To investigate the SCNs, four regions of interest (ROIs), representing seeds, were selected from the 135 preprocessed images. These following seed ROIs that anchor the DMN medial temporal subsystem (right entorhinal cortex [coordinates: 25,-9,-28]) [49], DMN midline core subsystem (left posterior cingulate cortex [PCC; coordinates: −2,-36, 35]) [50, 51], SN (right frontoinsular cortex [coordinates: 38, 26,-10]), and ECN (right dorsolateral prefrontal cortex [coordinates: 44, 36, 20]) [31] were selected (Fig. 1a). The laterality was based on the original report of the seeds. As the pathology or functional connectivity in typical patients with AD is distributed symmetrically, we did not perform a contralateral seed analysis in this study.

From the modified GM images, the GM volumes of a 4-mm radius sphere around the seed ROI coordinates were also calculated, followed by four separate correlation analyses using the extracted GM volumes as the covariates of interest. The two genotype groups were modeled separately. For each genotype group, specific contrasts were set to identify voxels that showed positive correlations for each seed ROI, forming the SCN. The results reflected the SCNs of each ROI and the threshold was set at *p* < 0.01, corrected for false discovery rate (FDR) with a cluster size >100 voxels.

In addition, to investigate how genetic variance may interfere with SCN clusters, voxels showing significant differences in the regression slopes in each seed-peak cluster correlations were compared, pointing to possible interactions between TT > C-carrier or TT < C-carrier based on the dosage-related IL-1 beta protein expression [13]. Specific t contrasts were established to map the voxels that expressed significant between-group associations. The threshold for the resulting statistical parametric maps was set at *p* < 0.001 (uncorrected) with a cluster size >100 voxels. For the peak clusters showing significant between-group differences, a 4-mm radius sphere was placed on the peak voxel, and the GM volumes were calculated for regression analysis. To evaluate the clinical significance of the seed or identified peak voxel, we used a linear regression model with the cognitive test scores serving as the dependent variable. The threshold was set at *p* < 0.05 with multiple corrections.

#### WM analysis

The fractional anisotropy (FA) maps were obtained using the functional MRI of the brain software library (FSL) version 4.0.1 package (http://www.fmrib.ox.ac.uk). A direct comparison of two genotype groups of the diffusion indices used permutation-based, non-parametric inference for the cluster size [52] and Randomise 2.0 software. A restrictive statistical threshold was used (the threshold-free, cluster-enhancement threshold with *p <* 0.05, corrected for multiple comparisons). To provide the between-group quantitative analysis, the WM parcellation algorithm [53] and calculation of 11 major bundles were performed. The FA from the each association bundle was extracted for group-interaction analysis or correlations with neurobehavior scores.

### Statistical analysis

Clinical and laboratory data were expressed as mean ± standard deviation. The student’s *t* test was used to compare levels of cerebrovascular risk biomarkers or continuous variables of the C-carriers and T homozygotes. All statistical analyses were conducted using SPSS software (SPSS version 13 for Windows®, SPSS Inc., Chicago, IL). Pearson correlation adjusted for possible confounders were perfomed to assess the associations between continuous variables. Statistical significance was set at *p* < 0.05.

## Results

### Demographic data, cognitive data, and NPI

The genotype distribution was in Hardy-Weinberg equilibrium (*χ* ^2^ = 0.28, *p* = 0.6) and the demographic characteristics, neuropsychiatric test or cerebrovascular risk biomarker results of the two genotype groups are shown in Table 1. The clinical dementia rating scores and genotype groups distributions were not significant (E4 genotype, *χ* ^2^ = 0.971, *p* = 0.262; IL-1 beta genotype, *χ* ^2^ = 2.682, *p* = 0.615).

**Table 1:** Demographical characteristics and neuropsychiatric tests in the interleukin-1 beta C-carrier and TT-carriers in 135 cases

Group	C-carrier (*n* = 108)	TT-carriers (*n* = 27)	*p* value
Age	71.7(14.2)	72.6(7.4)	0.77
Education (year)	6.9(4.7)	9.3(4.8)	0.02
Apolipoprotein E4 carrier (positive case, %)	42.6%	48.1%	0.67
Sex (male/female)	56/52	14/13	1
Mini-Mental State Examination	20.2(6.2)	20.6(7.7)	0.82
CASI total scores	67.8(20.2)	68.3(25.1)	0.89
CASI executive function test scores	25.3(7.9)	25.2(10.1)	0.97
CASI subdomains			
Short term memory	5.5(3.8)	5.4(3.8)	0.90
Orientation	12.8(5.1)	12.9(6.0)	0.88
Long term memory	8.3(2.4)	8.4(2.7)	0.90
Language	8.2(2.1)	8.1(2.8)	0.83
Drawing	7.8(2.8)	8.4(2.6)	0.37
Attention	6.3(1.4)	6.3(1.6)	0.98
Verbal fluency	5.2(2.5)	5.3(3.7)	0.86
Abstract thinking	8.2(2.8)	7.9(3.2)	0.64
Mental manipulation	5.6(3.2)	5.7(3.4)	0.92
Cerebrovascular risk biomarkers			
High sensitive C reactive protein (mg/L)	2.8(4.9)	2.4(5.2)	0.68
Homocysteine (umol/L)	12.9(4.3)	11.5(4.7)	0.14
Hemoglobin-A1C (%)	6.1(1.0)	6.2(1.7)	0.74
Creatinine (mg/dl)	0.9(0.4)	0.9(0.2)	0.52
High-density lipoprotein (mg/dl)	57.1(17.0)	61.0(16.6)	0.29
Low-density lipoprotein (mg/dl)	106.2(38.1)	97.4(28.6)	0.27
Total cholesterol (mg/dl)	188.5(40.8)	180.6(36.0)	0.36
Triglyceride (mg/dl)	120.7(62.0)	110.7(55.0)	0.44
Vitamin B12 (pg/dl)	589.1 (315.7)	687.2(428.1)	0.19
Folate (ng/dl)	12.0(5.6)	12.8(5.4)	0.52

Data are presented as mean (standard deviation) or number (percentage (%))

Attention, verbal fluency, abstract thinking, and mental manipulation sub-domain scores of the CASI were added to assess executive function

APOE4 carriers were defined as the presence of one or two APOE4 alleles

*CASI* Cognitive Ability Screening Instrument

### Patterns of SCN and genetic variants

A direct comparison between the GM volume of C-carriers or T homozygotes using voxel-based morphometry [54] showed no significant volumetric differences (with the threshold set at *p* < 0.05, corrected for a FDR with a cluster size >100 voxels).

According to the genotype classification (C-carriers and TT-carriers) and four seeds (Fig. 1a), there were no significant differences in the GM volumes of each seed (Fig. 1b). The SCN patterns and clusters for each genotype are shown in Fig. 1c and Additional file 1: Table S1–S8.

### Relationships between seed region volumes and cognitive scores

We first explored whether each seed region volume was correlated with the cognitive test scores in each group, adjusted for years of education (Table 2). The posterior cingulate, frontoinsular seed volume correlated variably with cognitive test scores in both genotypes, while the dorsolateral prefrontal seed showed significance only in the TT group.

**Table 2:** Correlation matrix between cognitive test scores with seed volume

Seed region	R entorhinal		L posterior cingulate		R frontoinsular		R dorsolateral prefrontal	
MNI coordinates	(25,-9,-28)		(−2,-36, 35)		(38, 26,-10)		(44, 36, 20)	
IL-1 beta genotypes	C-carrier	TT	C-carrier	TT	C-carrier	TT	C-carrier	TT
MMSE	0.189	−0.122	0.324**	0.451*	0.355**	0.464*	−0.039	0.489*
CASI total scores	0.140	−0.105	0.314**	0.391*	0.286**	0.393*	−0.027	0.530**
CASI EFT scores	0.109	−0.158	0.294**	0.344	0.219*	0.407*	0.000	0.510**
CASI Subdomains								
Short Term Memory	0.085	−0.012	0.308**	0.275	0.335**	0.475*	0.064	0.401*
Orientation	0.136	−0.133	0.308**	0.376	0.342**	0.362	−0.044	0.457*
Long Term Memory	0.188	0.074	0.172	0.351	0.148	0.131	−0.041	0.485*
Language	0.155	−0.101	0.290**	0.394*	0.235**	0.255	−0.057	0.484*
Drawing	0.045	−0.040	0.058	0.389*	0.054	0.281	−0.078	0.472*
Attention	−0.013	−0.174	0.200	0.376	0.246**	0.421*	0.077	0.511
Verbal fluency	0.083	0.050	0.272**	0.406*	0.186	0.455*	−0.001	0.349
Abstract thinking	0.288**	−0.298	0.174	0.291	0.200**	0.141	0.030	0.478*
Mental manipulation	−0.018	−0.028	0.285**	0.159	0.074	0.424*	−0.091	0.367

Numbers indicate Pearson correlation coefficients, ^*^ *p* < 0.05; **p < 0.01

*MMSE* Mini-Mental State Examination, *EFT* executive function test, *R* right, *L* left, *IL*-*1* interleukin-1, *CASI* Cognitive Abilities Screening Instrument, *MNI* Montreal Neurological Institute

### Peak clusters showing significant interactions between genotype groups

**Fig. 2:**
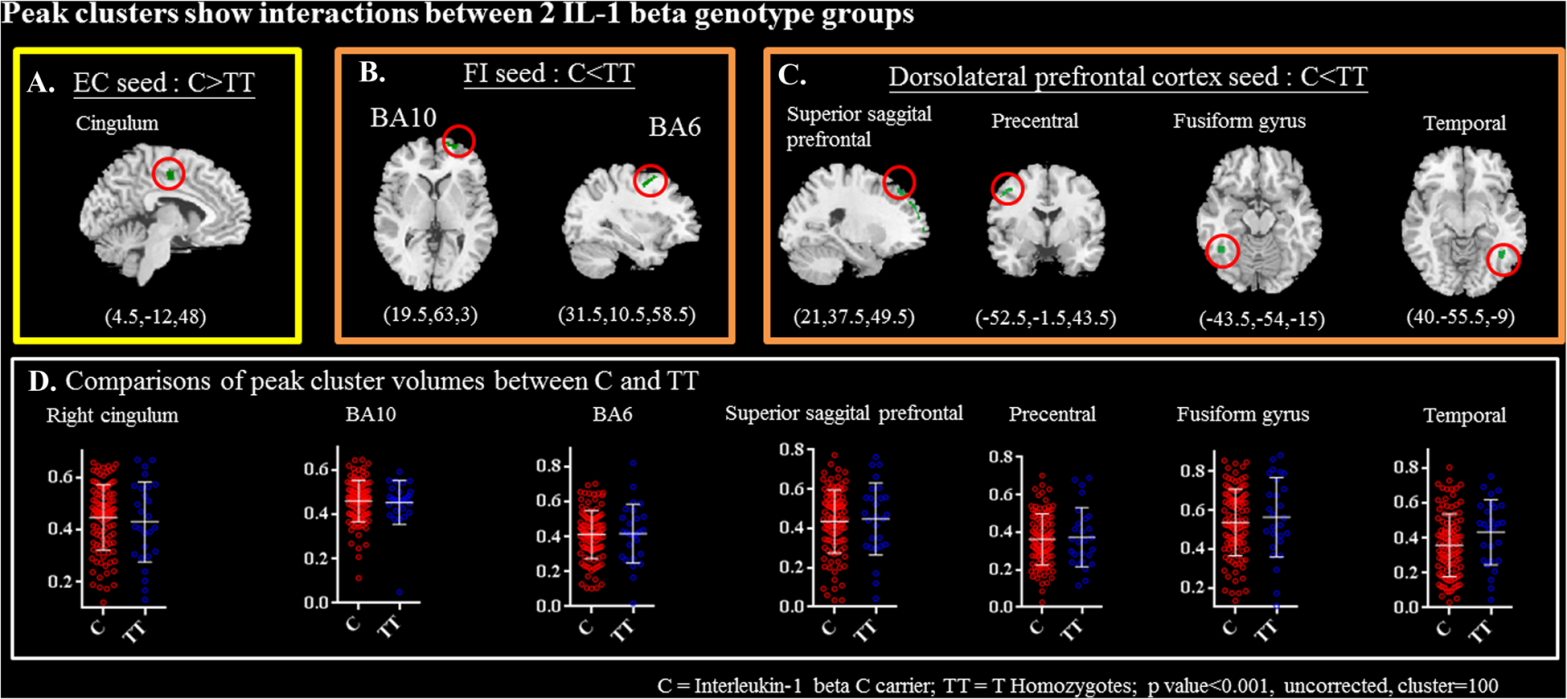
Peak clusters showing significant interactions of (**a**) C-carriers (=C) > T homozygotes from the entorhinal seed, (**b**) C-carriers < T homozygotes from the frontoinsular (FI) seed, or (**c**) C-carriers < T homozygotes from the dorsolateral prefrontal seed. (x,y,z) = Montreal Neurological Institute coordinates. (**d**) peak cluster volume comparisons (displayed as mean and standard deviation) showed no significant differences. Threshold set as *p* < 0.05

For each seed, we further explored the genotypic interactions with regards to the topography showing differences in structural covariance strength between seed and peak clusters (Fig. 2; Table 3).

**Table 3:** Connectivity interactions of interleukin-1 beta genotypes with pre-defined seed

Seed	Peak regions		Stereotaxic coordinates			Extent	MaxT	*p* value
			*x*	*y*	*z*			
C-carrier > TT								
Entorhinal seed	Middle cingulum	R	5	−12	48	118	3.81	0.0001
PCC seed	N.A							
Frontoinsular seed	N.A							
Dorsolateral prefrontal	N.A							
C-carrier < TT								
Entorhinal seed	N.A							
PCC seed	N.A							
Frontoinsular seed	BA 10	R	19.5	63	3.0	196	4.51	0.0001
	Middle frontal	R	31.5	10.5	58.5	243	3.47	0.0001
Dorsolateral prefrontal	Superior frontal	R	21	37.5	49.5	671	5.09	0.0001
	Precentral	L	−52.5	−1.5	−43.5	214	3.58	0.0001
	Fusiform	L	−43.5	−54	−15	123	4.17	0.0001
	Inferior temporal	R	40.5	−55.5	−9	119	4.03	0.0001

Peak regions are within the main cluster

Max T is the maximum T statistic for each local maximum. *p* < 0.001 with cluster size = 100

*N.A* not available, *PCC* Posterior cingulate cortex, *BA* Brodmann area, *R* right, *L* left

The middle cingulum that anchored to the entorhinal seed was the only significant cluster showing C-carriers > TT in covariance strength (Fig. 2a). In contrast, there were two clusters that anchored to the right frontoinsular seed (Fig. 2b) and four clusters that anchored to the right dorsolateral prefrontal cortex seed (Fig. 2c) showing TT > C-carriers in covariance strength. Meanwhile, direct comparisons of the peak clusters volume between two genotype groups showed no significant differences (Fig. 2d).

### Clinical significance of peak clusters showing genotype differences

The clinical significance of the aforementioned seven peak clusters showing genotype interactions was evaluated by partial correlation analysis with cognitive tests (Table 4). For right cingulum volume, the correlation analysis with cognitive test scores showed significance only in the C-carriers. For the six peak clusters showing TT > C-carriers in covariance strength, the frontoinsular seed- BA10 or -BA6 and dorsolateral-right temporal cluster showed significant correlations with cognitive tests in TT genotype. For C-carriers, the fusiform gyrus showed significant correlation with cognitive tests.

**Table 4:** Correlation matrix between cognitive test scores with peak clusters volume

Covariance strength relationship	C > TT	C < TT					
Seed region	R entorhinal	R frontoinsular seed		R dorsolateral seed			
Peak clusters	R cingulum	BA10	BA6	R superior sagittal prefrontal	L precentral	L fusiform gyrus	R temporal
Cognitive tests	IL-1 beta genotype :-511 C-carriers						
MMSE	0.24*	0.07	0.15	0.16	0.24*	0.17	0.06
CASI total scores	0.26**	0.09	0.19*	0.17	0.28**	0.19	0.05
CASI EFT scores	0.21*	0.12	0.16	0.13	0.25**	0.19*	0.07
CASI subdomains							
Short-term memory	0.21*	0.04	0.08	0.12	0.26**	0.07	0.05
Orientation	0.22*	−0.03	0.15	0.09	0.24*	0.16	0.08
Long-term memory	0.31**	0.05	0.16	0.18	0.25**	0.04	0.15
Language	0.16	0.04	0.16	0.30**	0.25	0.09	−0.06
Drawing	0.14	0.16	0.19	0.11	0.05	0.29**	−0.06
Attention	0.17	0.05	0.17	0.06	0.30**	0.01	−0.07
Verbal fluency	0.23*	0.04	0.04	0.18	0.23*	0.18	0.03
Abstract thinking	0.20*	0.11	0.12	0.09	0.12	0.25*	0.07
Mental manipulation	0.07	0.14	0.14	0.12	0.17	0.17	0.15
Cognitive tests	IL-1 beta genotype :-511 TT						
MMSE	0.12	0.43*	0.39*	0.36	0.30	0.40*	0.48*
CASI total scores	0.14	0.47*	0.44*	0.48*	0.34	0.35	0.56**
CASI EFT scores	0.21	0.44*	0.39*	0.39*	0.40*	0.35	0.60**
CASI Subdomains							
Short Term Memory	−0.03	0.34	0.19	0.38	0.02	0.37	0.22
Orientation	0.16	0.43*	0.39	0.42*	0.24	0.30	0.46*
Long Term Memory	0.02	0.36	0.49	0.54**	0.39*	0.22	0.56**
Language	0.11	0.38	0.51	0.48*	0.43*	0.30	0.60**
Drawing	0.09	0.55**	0.48	0.54**	0.31	0.26	0.51**
Attention	0.06	0.32	0.27	0.35	0.39	0.33	0.57**
Verbal fluency	0.30	0.69**	0.46*	0.27	0.29	0.23	0.39*
Abstract thinking	0.26	0.37	0.40*	0.39	0.41*	0.19	0.56**
Mental manipulation	0.01	0.29	0.30	0.29	0.28	0.43*	0.47*

Numbers indicate Pearson correlation coefficients between peak cluster volume and test scores, *p < 0.05; **p < 0.01

*MMSE* Mini-Mental State Examination, *EFT* executive function test, *R* right, *L* left, *IL*-*1* interleukin-1, *CASI* Cognitive Abilities Screening Instrument, *BA* Brodmann area, *C* IL-1 beta C-carrier, *TT* T Homozygotes

### No differences of WM integrities between two genotype groups

For major WM tract FA, the differences between the two genotype groups and correlations with cognitive tests are shown in Table 5. The correlation between fiber bundle FA and cognitive tests were significant in both groups but there were no statistic differences between the two genotype groups.

**Table 5:** Significant relationships between major white matter tracts integrity and interleukin-1 beta genotype groups or cognitive test scores

	Fractional anisotropy		MMSE		CASI total scores		CASI EFT	
Major white matter tract fractional anisotropy	C-carrier	TT	C-carrier	TT	C-carrier	TT	C-carrier	TT
Forceps major	0.47(0.04)	0.48(0.05)	0.25*	0.40*	0.29**	0.34	0.35**	0.40*
Forceps minor	0.35(0.03)	0.35(0.03)	0.26*	0.48*	0.28**	0.44*	0.34**	0.52*
Anterior thalamic radiation	0.60(0.06)	0.61(0.06)	0.22*	0.48*	0.24*	0.42*	0.30**	0.48*
Corticospinal tract	0.99(0.06)	0.99(0.06)	0.37**	0.10	0.39**	0.07	0.44**	0.17
Cingulum	0.68(0.06)	0.68(0.07)	0.29**	0.41*	0.32**	0.36	0.32**	0.43*
Cingulum (hippocampus)	0.58(0.07)	0.59(0.06)	0.41**	0.18	0.45**	0.16	0.38**	0.17
Inferior fronto-occipital fasciculus	0.74(0.05)	0.74(0.06)	0.22*	0.29	0.26*	0.24	0.34**	0.29
Inferior longitudinal fasciculus	0.74(0.05)	0.75(0.05)	0.26*	0.31	0.30**	0.29	0.37**	0.32
Superior longitudinal fasciculus	0.63(0.05)	0.63(0.06)	0.20*	0.24	0.23*	0.22	0.27**	0.29
Uncinate fasciculus	0.68(0.05)	0.68(0.04)	0.26*	0.20	0.28**	0.13	0.30**	0.17
Superior longitudinal fasciculus (temporal part)	0.90(0.08)	0.89(0.09)	0.14	0.26	0.18	0.27	0.24*	0.32

Data of Fractional Anisotropy represent mean (standard deviation), *MMSE* Mini-Mental State Examination scores, *CASI* Cognitive Ability Screening Instrument, *EFT* executive function test, *EFT* Sum of attention, verbal fluency, abstract thinking, and mental manipulation sub-domain scores of the CASI, *TT* T homozygotes

Numbers indicate mean and standard deviation or Pearson correlation coefficient; *p < 0.05 **p < 0.01

## Discussion

This study provides data on the network-specific genotype influence of IL-1 beta in the early stages of AD. There were three main findings. First, the seed-based SCN pattern validated the hypothesis that IL-1 beta C-511 T polymorphism targets different functional networks in patients with AD, of which interactions between C-carriers and T homozygotes differed in covariance strength. The increased covariance strength between the frontoinsular-BA10, frontoinsular-BA6, and dorsolateral-temporal axis in TT homozygotes suggest increased influences involving the anterior prefrontal-related degenerative scaffold by this genotype. In contrast, the entorhinal-cingulum connections were increased in the C-carriers. Second, IL-1 beta C-511 T functional polymorphism exerts no significant effects on the preselected cerebrovascular risk biomarkers, seed, or peak cluster volumes. Finally, the C-511 T genetic effect may interfere with the SCN network independent of the WMHs, as no differences were found between the two genotype groups in diffusion parameters.

### IL-1 beta genotypes and AD diagnosis

The impact of IL-1 beta C-511 T polymorphism on amyloid beta immunoreactivity in the brains of patients with AD [55] or amyloid beta levels in the cerebral spinal fluid of patients with AD [56] suggest the important role of this polymorphism in modulating the pathology of AD, although findings regarding C-511 T polymorphism on the susceptibility to AD or the effect on AD onset age have been inconsistent [14, 55–58]. IL-1 beta exerts a myriad of effects in the brain and, in particular, plays a significant role in hippocampal synaptic function, which is implicated in the AD pathogenesis [9]. Although an increased IL-1 beta expression has been reported in Taiwan Chinese TT-carriers [14] which is thought to modulate hippocampal function, we did not find such relationships between the −511 TT polymorphism and entorhinal seed volume and cognitive outcomes. In contrast, the significant correlations between cognitive test scores and cingulum volume (i.e., entorhinal-interconnect peak cluster) in the −511 C-carriers may support the selective IL-1 beta genetic modulation in the entorhinal anchored network of AD patients. In contrast, no association was reported between the IL-1 −511 TT polymorphism and AD in Hong Kong [59] or China [60] Han Chinese. This discrepancy may partially reflect differences in ethnicity among Han Chinese [61].

While the DMN in patients with AD has been demonstrated to show reduced connectivity compared with controls, enhanced resting-state functional connectivity of the SN [62] or ECN [32, 33] has also been found in AD patients. The increase in network connectivity in AD has been hypothesized to reflect a compensatory mechanism for weakened posterior hubs [63] and to participate in functions such as sustained attention, working memory, response selection, or suppression [31]. As such, changes in structural covariance strength in the IL-1 beta C-511 T polymorphism may infer the capacity of this polymorphism to modulate symptoms in patients with AD. As increased covariance strength indicates stronger interconnections between seed and peak clusters, we speculate the −511 TT variant may potentially be a more vulnerable genotype group in this context. Once the compensatory regions located in the anterior brain axis were involved by pathology, the specific involvement of the SN and ECN in the TT group may accelerate the degeneration process.

### IL-1 beta genotypes and brain network analysis

The mechanism by which IL-1 beta C-511 T polymorphism affect the brain networks in patients with AD has yet to be fully established. The higher IL-1 beta gene expression during long-term potentiation suggests its role in synaptic function [15]. In patients with mild to moderate stage AD, carriers of the -511C allele have been shown to have a reduced inflammatory response, and this has been proposed to allow for better cognitive improvements on a ketone body based treatment [64]. Studies of other IL-1 beta polymorphisms have also suggested the anti-correlated relationships between the expression of IL-1 beta and cognitive performance [65]. Functional connectivity analysis in normal elderly has shown that the IL-1 beta −511 TT carriers have reduced connectivity of the anterior mid-cingulate-prefrontal-striate networks [66], pointing to the role of this functional polymorphism and the anterior brain network.

Our analysis provides evidence that GM network alterations may be considered as the endophenotype of the IL-1 beta C-511 T polymorphism that predicts the cognitive outcomes. Although we purposed that increased structural covariance strength within functional network may lead to detrimental effects in patients with AD, whether the genetic effects by our reports fully address the hippocampal IL-1 beta expression still left unexplored. Data on IL-1 beta genotypes and postmortem IL-1 beta level examinations are needed to elucidate the consequences. In line with our findings, several neuroimaging studies have also demonstrated the detrimental effects of IL-1 beta -511 T allele on fronto-temporal GM [67, 68], left dorsolateral prefrontal cortex [68–70], or parahippocampus [71] in various brain disorders.

Increased structural covariance between the cingulum peak cluster and entorhinal seed was found in the C-carriers compared with TT homozygotes. Adjusted for educational years, this covariance also determines cognitive test scores in the C carriers, suggesting the unique covariance strength relationship in terms of spatial distribution in AD. Current research criteria of AD [40] do not include the influence of genomic data; however, recent results from studies on neuronal exosomes and nanosomes [72–74] support the importance of metabolic and inflammatory abnormalities in predicting the prodromal phase. Since metabolic and inflammatory functions are greatly under genetic control, this may be productive if genetic profiling is taken into consideration in the diagnosis of patients with cognitive deficits.

### IL-1 beta genotypes and cognition

There have been many IL-1 beta genetic association studies on individuals without dementia during the cognitive aging stages. A cross-sectional study reported a significant relationship between C-511 T polymorphism and episodic memory, with a better performance in the C homozygous than in the CT/TT group [75]. As the authors [75] did not find any association between this polymorphism and attention, processing or motor function, they suggested that the effect of this IL-1 beta polymorphism on cognition may be domain-specific (i.e., memory-specific). In accordance with their findings, two other studies on elderly males [45] and elderly females [76] showed that −511 C-carriers had higher scores in general or in selective cognitive tests compared to the T homozygotes. After adjusting for years of education, our correlation results suggested parallel relationships between seed and peak cluster volumes in relation to cognitive tests scores in the genotype groups. As the cross-sectional cognitive test design may have resulted in population selections bias, data on cross-sectional models may not fully address whether IL-1 beta C-511 T polymorphisms mediate domain-specific cognitive deficits or whether they are more generalized [45, 77, 78]. A longitudinal follow-up study may increase the accuracy of genotypes and involved cognitive domains. In contrast to previous reports, our study analysis did not establish a direct C-511 T genotype effect on cognitive test scores.

### IL-1 beta genotypes and WMHs

Homozygotes for the IL-1 beta -511 T allele have been associated with larger WMHs than other genotypic combinations in the elderly without dementia [24]; however, we did not find such an association in AD. Nonetheless, our results show that the IL-1 beta genetic effect can be solely present in the GM network, and that this effect is independent of the modulation of WMHs. Our study only enrolls patients in the early stage and AD pathology is more localized in the GM in the early stage of AD. Thus, the influence of IL-1 beta C-511 T polymorphism on WMHs may be stage-specific.

### Study limitations

An important limitation of this study is that we did not include a control group. The enrolment of controls may help to understand whether IL-1 beta C-511 T polymorphism has similar effects on the normative brain network. Nonetheless, the aim of this study was to determine whether changes in the SCN caused by IL-1 beta genetic polymorphism in AD patients. Our results support the data from elderly healthy subjects [71] that genetic variations of IL-1 beta mediate the dorsolateral prefrontal cortex, yet the analysis of SCN patterns with changes in structural covariance strength was not available. Another potential limitation is that we reported the genetic effect between the C-carriers and TT homozygotes, and such group stratification could not explore the model of heterozygote advantage. We based this decision on the small sample size and a previously published report [13] on the IL-1 beta dosage effects. Thirdly, as clinical significance was established in four pre-defined networks, we did not test whether other networks participated in genetic modulation. The use of independent component analysis [79] or resting state functional MRI data may elucidate other potential networks and also validate the findings observed in this study. Finally, cognitive tests such as MMSE and CASI are often used as screening tests. Further studies are warranted that include an extensive battery of tests.

## Conclusions

In the early stages of AD, our analysis supports that IL-1 beta C-511 T polymorphism modulates the strength of the structural covariance independent of WMHs. The −511 TT homozygotes convey increased covariance patterns in anterior brain networks that may then modulate the degenerative process differently from the patterns of entorhinal-cingulum networks in C-carriers.

## Additional file

Additional file 1: The peak clusters in the structural covariance network. (DOCX 39 kb)

## Abbreviations

AD: Alzheimer’s disease
CASI: Cognitive Abilities Screening Instrument
DARTEL: Diffeomorphic anatomical registration using exponentiated lie algebra
DMN: Default mode network
ECN: Executive control network
EFT: Executive function test
FA: Fractional anisotropy
FDR: False discovery rate
GM: Gray matter
IL-1: Interleukin-1
MMSE: Mini-Mental State Examination
MNI: Montreal Neurological Institute
PCR: Polymerase chain reaction
ROIs: Regions of interest
SCNs: Structural covariance networks
SN: Salience network
WM: White matter
WMHs: White matter hyperintensities
