# Supplementary material for: Genetic effect of interleukin-1 beta (C-511T) polymorphism on the structural covariance network and white matter integrity in Alzheimer’s disease
Source: J Neuroinflammation. 2017 Jan 18;14:12. doi: 10.1186/s12974-017-0791-z (PMC5242022; doi:10.1186/s12974-017-0791-z)
Supplement: Additional file 1: — The peak clusters in the structural covariance network. (DOCX 39 kb) [file 12974_2017_791_MOESM1_ESM.docx]

**Table S1** Structural covariance network for Interleukin-1 beta -511C carrier with right entorhinal cortex as seed.

| **Main Cluster** | **Peak regions** |  | **Stereotaxic coordinates** | | | **Extent** | **Max T** | **P-value** |
| --- | --- | --- | --- | --- | --- | --- | --- | --- |
|  |  | Side | x | y | z |  |  |  |
| ParaHippocampal |  | R | 26 | -12 | -27 | 4240 | 16 | 0.000 |
|  | Fusiform | R | 32 | -4 | -44 | s.c | 4.81 | 0.000 |
|  | Inferior Temporal | R | 44 | -11 | -33 | s.c | 4.5 | 0.000 |
| ParaHippocampal |  | L | -23 | -22 | -29 | 871 | 5.66 | 0.000 |
|  | ParaHippocampal | L | -20 | -12 | -32 | s.c | 5.56 | 0.000 |
| Cerebelum_Crus1 |  | R | 33 | -51 | -38 | 392 | 4.8 | 0.000 |
|  | Cerebelum_6 | R | 30 | -45 | -33 | s.c | 4.34 | 0.000 |
| Middle Cingulum |  | R | 8 | 35 | 36 | 149 | 4.59 | 0.000 |
|  | Superior medial Frontal | R | 6 | 24 | 43 | s.c | 4.36 | 0.000 |
| Superior Temporal |  | R | 56 | -3 | -12 | 152 | 3.96 | 0.000 |
|  | Middle Temporal | R | 57 | -7 | -20 | s.c | 3.93 | 0.000 |

Peak regions are within the Main cluster

Max T is the maximum T statistic for each local maximum. P<0.05 based on non-stationary cluster-extent False discovery rate correction. s.c: same clusters

**Table S2** Structural covariance network for Interleukin-1 beta -511T homozygotes with right entorhinal cortex as seed.

| **Main Cluster** | **Peak regions** |  | **Stereotaxic coordinates** | | | **Extent** | **Max T** | **P-value** |
| --- | --- | --- | --- | --- | --- | --- | --- | --- |
|  |  | Side | x | y | z |  |  |  |
| ParaHippocampal |  | R | 26 | -12 | -27 | 168 | 11.88 | 0.000 |

Peak regions are within the Main cluster

Max T is the maximum T statistic for each local maximum. P<0.05 based on non-stationary cluster-extent False discovery rate correction. s.c: same clusters

**Table S3** Structural covariance network for Interleukin-1 beta -511C carrier with left posterior cingulate cortex as seed.

| **Main Cluster** | **Peak regions** |  | **Stereotaxic coordinates** | | | **Extent** | **Max T** | **P-value** |
| --- | --- | --- | --- | --- | --- | --- | --- | --- |
|  |  | Side | x | y | z |  |  |  |
| Middle Cingulum |  | L | -2 | -36 | 34 | 182692 | 26.99 | 0.000 |
|  | Middle Cingulum | L | -2 | -15 | 40 | s.c | 12 | 0.000 |
|  | Middle Temporal | L | -56 | -60 | 16 | s.c | 9.43 | 0.000 |

Peak regions are within the Main cluster

Max T is the maximum T statistic for each local maximum. P<0.05 based on non-stationary cluster-extent False discovery rate correction. s.c: same clusters

**Table S4** Structural covariance network for Interleukin-1 beta -511T homozygotes with left posterior cingulate cortex as seed.

| **Main Cluster** | **Peak regions** |  | **Stereotaxic coordinates** | | | **Extent** | **Max T** | **P-value** |
| --- | --- | --- | --- | --- | --- | --- | --- | --- |
|  |  | Side | x | y | z |  |  |  |
| Middle Cingulum |  | L | -2 | -36 | 36 | 4551 | 15.9 | 0.000 |
|  | Precuneus | R | 12 | -55 | 25 | s.c | 6.48 | 0.000 |
|  | Paracentral Lobule | L | -6 | -30 | 52 | s.c | 5.5 | 0.000 |
| Middle Temporal |  | R | 62 | -8 | -26 | 865 | 6.48 | 0.000 |
|  | Middle Temporal | R | 63 | -18 | -9 | s.c | 5.08 | 0.000 |
|  | Middle Temporal Pole | R | 56 | 8 | -30 | s.c | 4.85 | 0.000 |
| Inferior Occipital |  | L | -45 | -75 | -8 | 437 | 6.19 | 0.000 |
|  | Inferior Temporal | L | -53 | -60 | -6 | s.c | 4.79 | 0.000 |
| Precentral |  | R | 41 | 3 | 48 | 329 | 5.82 | 0.000 |
|  | Middle Frontal | R | 35 | 20 | 49 | s.c | 5.76 | 0.000 |
|  | Middle Frontal | R | 39 | 12 | 46 | s.c | 4.3 | 0.000 |
| Angular |  | R | 50 | -63 | 24 | 360 | 5.61 | 0.000 |
|  | Angular | R | 42 | -61 | 40 | s.c | 5.13 | 0.000 |
|  | Inferior Parietal | R | 56 | -60 | 37 | s.c | 4.51 | 0.000 |
| Inferior Temporal |  | L | -44 | -33 | -29 | 411 | 5.49 | 0.000 |
|  | Inferior Temporal | L | -48 | -46 | -20 | s.c | 5.12 | 0.000 |
|  | Inferior Temporal | L | -56 | -48 | -15 | s.c | 4.86 | 0.000 |
| SupraMarginal |  | R | -56 | -37 | 31 | 203 | 5.46 | 0.000 |
|  | Inferior Parietal | L | -57 | -43 | 40 | s.c | 4.9 | 0.000 |

Peak regions are within the Main cluster

Max T is the maximum T statistic for each local maximum. P<0.05 based on non-stationary cluster-extent False discovery rate correction. s.c: same clusters

**Table S5** Structural covariance network for Interleukin-1 beta -511C carrier with right frontoinsular seed.

| **Main Cluster** | **Peak regions** |  | **Stereotaxic coordinates** | | | **Extent** | **Max T** | **P-value** |
| --- | --- | --- | --- | --- | --- | --- | --- | --- |
|  |  | Side | x | y | z |  |  |  |
| Inferior orbital Frontal |  | R | 36 | 26 | -11 | 121161 | 18.14 | 0.000 |
|  | Middle Temporal | L | -59 | -21 | -6 | s.c | 7.91 | 0.000 |
|  | Insula | R | 42 | 11 | 4 | s.c | 7.28 | 0.000 |
| Postcentral |  | L | -33 | -27 | 51 | 313 | 3.9 | 0.000 |
| Precentral |  | R | 24 | -21 | 60 | 152 | 3.7 | 0.000 |
|  | Precentral | R | 32 | -22 | 52 | s.c | 3.06 | 0.001 |
| Superior Parietal |  | L | -30 | -46 | 61 | 127 | 3.4 | 0.000 |

Peak regions are within the Main cluster

Max T is the maximum T statistic for each local maximum. P<0.05 based on non-stationary cluster-extent False discovery rate correction. s.c: same clusters

**Table S6** Structural covariance network for Interleukin-1 beta -511T homozygotes with right frontoinsular seed.

| **Main Cluster** | **Peak regions** |  | **Stereotaxic coordinates** | | | **Extent** | **Max T** | **P-value** |
| --- | --- | --- | --- | --- | --- | --- | --- | --- |
|  |  | Side | x | y | z |  |  |  |
| Inferior orbital Frontal |  | R | 38 | 27 | -12 | 117 | 11.97 | 0.000 |
| Posterior cingulum |  | R | 8 | -49 | 28 | 554 | 7.5 | 0.000 |
|  | Precuneus | R | 3 | -52 | 19 | s.c | 7.26 | 0.000 |
|  | Middle Cingulum | R | 6 | -33 | 39 | s.c | 6.2 | 0.000 |
| Superior Frontal |  | R | 20 | 60 | 16 | 292 | 6.84 | 0.000 |
|  | Superior Frontal | R | 23 | 63 | 0 | s.c | 6.71 | 0.000 |
|  | Superior medial Frontal | R | 9 | 66 | 0 | s.c | 5.61 | 0.000 |
| SupraMarginal |  | R | 57 | -34 | 45 | 165 | 6.58 | 0.000 |
|  | SupraMarginal | R | 56 | -45 | 40 | s.c | 5.93 | 0.000 |

Peak regions are within the Main cluster

Max T is the maximum T statistic for each local maximum. P<0.05 based on non-stationary cluster-extent False discovery rate correction. s.c: same clusters

**Table S7** Structural covariance network for Interleukin-1 beta C carrier with right dorsolateral prefrontal seed.

| **Main Cluster** | **Peak regions** |  | **Stereotaxic coordinates** | | | **Extent** | **Max T** | **P-value** |
| --- | --- | --- | --- | --- | --- | --- | --- | --- |
|  |  | Side | x | y | z |  |  |  |
| Middle Frontal |  | R | 42 | 36 | 19 | 5323 | 19.3 | 0.000 |
|  | Superior Frontal | R | 24 | 41 | 31 | s.c | 5.7 | 0.000 |
|  | Middle Frontal | R | 39 | 50 | 12 | s.c | 5.54 | 0.000 |
| Inferior orbital Frontal |  | L | -48 | 33 | -9 | 419 | 5.36 | 0.000 |
| Rolandic Operculum |  | L | -50 | 2 | 1 | 1018 | 5.05 | 0.000 |
|  | Superior Temporal | L | -50 | -22 | 3 | s.c | 4.65 | 0.000 |
|  | Superior Temporal | L | -44 | -31 | 9 | s.c | 4 | 0.000 |
| Frontal inferio triangular region |  | L | -42 | 30 | 25 | 742 | 4.88 | 0.000 |
|  | Middle Frontal | L | -29 | 39 | 28 | s.c | 4.44 | 0.000 |
|  | Frontal inferio triangular region | L | -41 | 42 | 10 | s.c | 4.25 | 0.000 |
| Middle Frontal |  | L | -27 | 27 | 40 | 124 | 4.75 | 0.000 |
| Frontal inferior operculum |  | L | -48 | 6 | 24 | 207 | 4.68 | 0.000 |

Peak regions are within the Main cluster

Max T is the maximum T statistic for each local maximum. P<0.05 based on non-stationary cluster-extent False discovery rate correction. s.c: same clusters

**Table S8** Structural covariance network for Interleukin-1 beta -511T homozygotes with right dorsolateral prefrontal seed.

| **Main Cluster** | **Peak regions** |  | **Stereotaxic coordinates** | | | **Extent** | **Max T** | **P-value** |
| --- | --- | --- | --- | --- | --- | --- | --- | --- |
|  |  | Side | x | y | z |  |  |  |
| Middle Frontal |  | R | 42 | 36 | 19 | 5729 | 8.24 | 0.000 |
|  | Superior Frontal | R | 17 | 41 | 45 | s.c | 7.3 | 0.000 |
|  | Superior Frontal | R | 15 | 50 | 33 | s.c | 6.69 | 0.000 |
| Insula |  | L | -42 | 14 | 4 | 4004 | 6.77 | 0.000 |
|  | Middle Frontal | L | -41 | 26 | 31 | s.c | 5.81 | 0.000 |
|  | Superior Frontal | L | -18 | 60 | 15 | s.c | 5.61 | 0.000 |
| Superior medial Frontal |  | L | -8 | 17 | 42 | 119 | 5.63 | 0.000 |
| Middle Cingulum |  | R | 8 | -31 | 40 | 251 | 5.58 | 0.000 |
| Precentral |  | L | -53 | 0 | 43 | 772 | 5.06 | 0.000 |
|  | Middle Frontal | L | -33 | 5 | 55 | s.c | 4.59 | 0.000 |
|  | Precentral | L | -42 | 5 | 48 | s.c | 4.59 | 0.000 |
| Postcentral |  | R | 30 | -43 | 69 | 123 | 4.99 | 0.000 |
|  | Postcentral | R | 39 | -40 | 62 | s.c | 4.76 | 0.000 |
| Midbrain |  | R | 3 | -19 | -2 | 333 | 4.62 | 0.000 |
|  | Thalamus | L | -6 | -28 | 1 | s.c | 4.22 | 0.000 |
| Inferior Tempora |  | R | 42 | -54 | -12 | 160 | 4.37 | 0.000 |
| Inferior orbital Frontal |  | R | 36 | 24 | -14 | 116 | 4.36 | 0.000 |
|  | Insula | R | 36 | 15 | -14 | s.c | 3.62 | 0.001 |

Peak regions are within the Main cluster

Max T is the maximum T statistic for each local maximum. P<0.05 based on non-stationary cluster-extent False discovery rate correction. s.c: same clusters
